# Monofocal intraocular lens based on the Bessel principle for improved intermediate vision: a comparative assessment

**DOI:** 10.1186/s12886-026-04973-9

**Published:** 2026-06-13

**Authors:** Frederick Kremser, Weijia Yan, Ramin Khoramnia, Gerd Uwe Auffarth, Grzegorz Łabuz

**Affiliations:** 1 The David J. Apple International Laboratory for Ocular Pathology, Department of Ophthalmology, Ruprecht-Karls-University of Heidelberg, Im Neuenheimer Feld 400, 69120 Heidelberg, Germany; 2 Eye Center, The Second Affiliated Hospital, School of Medicine, Zhejiang University, Hangzhou, China; 3 Department of Ophthalmology, Faculty of Medicine, University Hospital Carl Gustav Carus, TU Dresden, Dresden, Germany

**Keywords:** Enhanced monofocal, Monofocal-plus, Intraocular lens, Bessel principle

## Abstract

**Background:**

To compare the optical qualities of a novel enhanced monofocal intraocular lens (IOL) - Extend (Hanita), against a well-established IOL - Eyhance (ICB00, J&J Vision) - through in vitro benchmarking.

**Methods:**

Optical benchmarking was conducted using the OptiSpheric IOL PRO2 device in compliance with International Organization for Standardization (ISO) guidelines. Optical quality was assessed using the modulation transfer function (MTF), point spread function (PSF), area under the MTF (MTFa), and 1951 USAF test charts. Measurements were taken at 3.0 mm and 4.5 mm apertures using a model cornea that induced 0.13 μm of spherical aberration to simulate clinical performance.

**Results:**

Both IOLs demonstrated comparable optical performance. At a 3 mm aperture, the Extend produced an average MTF at 50 lp/mm of 0.38 ± 0.01, while the ICB00 produced 0.36 ± 0.01. The MTFa and derived logMAR visual acuity (VA) curves showed nearly complete overlap for defocus levels less than − 1.25 D, with both lenses yielding identical VA at far focus (-0.09 logMAR). At higher defocus levels, the observed difference between the models accounted for < 0.02 logMAR, which is small and below the resolution threshold of standard VA testing. Point spread function assessments revealed comparable light distribution for both IOLs.

**Conclusion:**

Under laboratory conditions, the Hanita Extend IOL shows comparable results to the Eyhance ICB00 regarding MTF function, USAF-Chart images, and PSF assessment.

## Introduction

Conventional monofocal intraocular lenses (IOLs) remain the most popular choice in cataract surgery today, offering excellent vision for a single focal point, though necessitating dependence on reading glasses [1]. However recently, there has been a noticeable transition towards monofocal+ (or enhanced monofocal) IOLs, with some even arguing that monofocal+ IOLs should become standard of care [2, 3]. These lenses maintain the high visual quality of distance vision found in standard models, while enabling a broader range of focus [4]. Crucially, this lessened dependence on glasses is achieved without increasing the risk of photic phenomena or compromising contrast sensitivity, as has been shown in many studies [4–6]. The same studies quantify the increase of intermediate VA as approximately 0.1 logMAR at 1.5 to 2D vergence [3, 6–8].

This gain of function is achieved through higher order aspheric designs or diffractive optics, but placed over a small pupil area, thus explaining the standard monofocal levels of halo, glare and contrast sensitivity [4, 9, 10].

Within this category, the TECNIS Eyhance (DIB00/ICB00) (Johnson & Johnson Surgical Vision, Inc., Santa Ana, CA, USA) occupies a prominent position. Although not the first enhanced monofocal IOL introduced, it has become one of the most frequently implanted models. This widespread adoption is underpinned by extensive laboratory and clinical research, which has provided an in-depth understanding of the lens’s capabilities [5, 6, 11–15]. Due to this robust volume of high-quality data, the Eyhance is now widely selected as the reference benchmark against which other emerging lenses are evaluated [16–18]. In terms of terminology it is important to note that DIB00 is the term for the preloaded version of the same lens, which is why in our study we refer to it solely as the ICB00.

A novel entrant in this category is the Hanita Extend (Hanita Lenses R.C.A Ltd., Kibbutz, Israel). This IOL utilizes a Bessel beam generation function to achieve an elongated depth of focus, with additional optimization for intermediate vision. Specifically, the lens employs a conical axicon surface to create this elongation. While Bessel beam technology is frequently used in engineering for laser material treatment, its application here represents a distinct optical approach.

Due to the current lack of scientific literature on the Hanita Extend, this study was conducted to compare the optical qualities of this new lens against the well-established Eyhance. We believe that this serves as the ideal candidate for in vitro benchmarking to provide valuable insights into the potential clinical optical performance of the Hanita Extend.

## Materials and methods

### Intraocular lenses

We studied two distinct monofocal IOLs, each having the same refractive power of + 20 D, designed to provide an extended depth of focus:

The Tecnis Eyhance (ICB00) is a hydrophobic acrylic lens that possesses a refractive index of 1.47 at 35 °C and an Abbe number of 55. Structurally, it features a continuous 360° posterior square edge (ProTEC frosted) with the haptics slightly shifted posteriorly relative to the central optic of the lens. The optical design incorporates a higher-order aspheric anterior surface that generates continuous power progression. The IOL also corrects a spherical aberration (SA) of 0.27 μm at 6 mm.

**Fig. 1:**
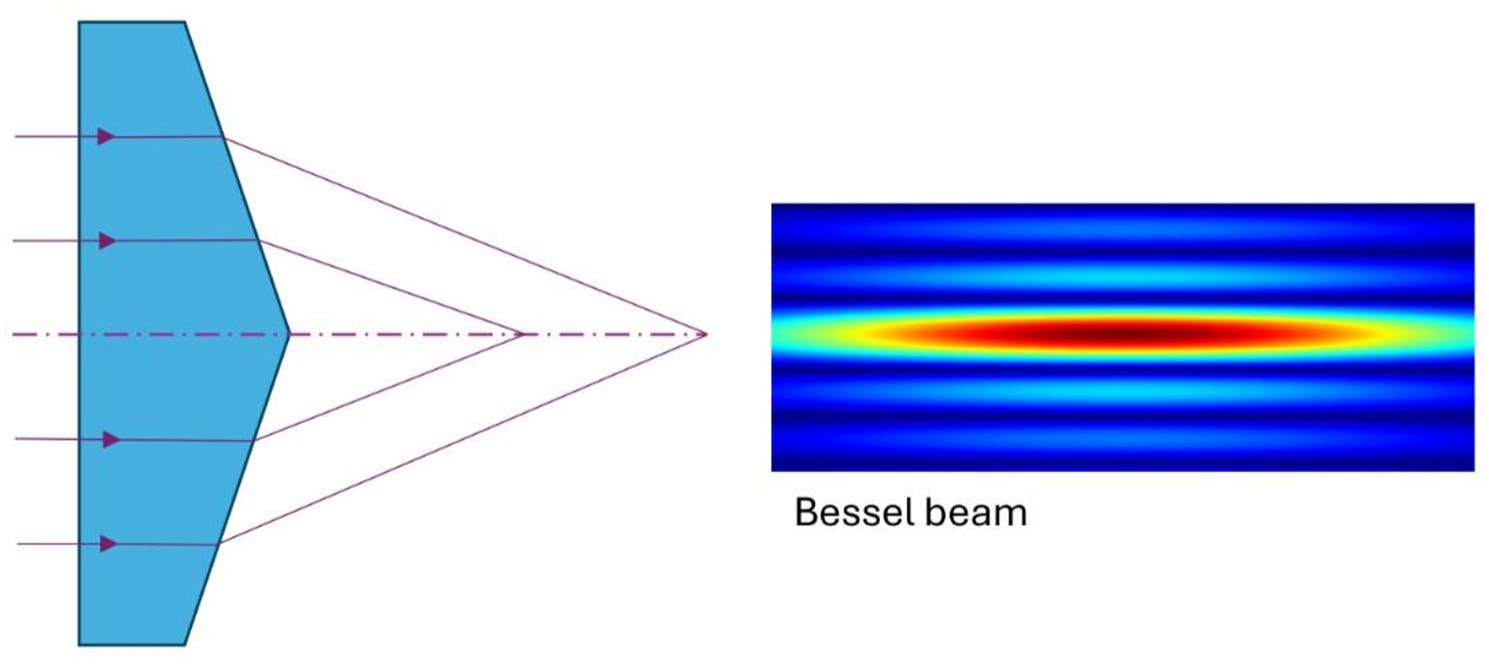
The visualization of the conical axicon surface providing an elongated DOF with an additional optimization for intermediate vision. The Bessel beam generation function elongates the DOF. In diameters larger than 3.5 mm, there is an aspheric surface optimization giving maximal energy to far vision for large apertures

The Hanita Extend is composed of a glistening-free hydrophobic acrylic material with a 3% water content, a refractive index of 1.48 (at 35 °C), and an Abbe number of 49. It features a spherical anterior surface and an aspheric posterior surface. The Hanita Extend corrects 0.13 μm of SA [19]. The optical design of the Extend IOL features a continuous, two‑zone refractive surface that implements the Bessel principle. The central zone incorporates an axicon‑based phase profile that transitions into a standard aspheric peripheral zone (Fig. 1). However, due to the finite aperture of an IOL, the resulting beam cannot be a mathematically ideal, non‑diffracting Bessel beam of infinite extent. Instead, this axicon‑based central zone produces a finite‑length, ‘Bessel‑like’ propagation region. Within this region, the axial intensity distribution remains relatively invariant compared to conventional Gaussian focusing.

### Optical metrology device and procedures

Optical benchmarking was conducted using the OptiSpheric IOL PRO2 (Trioptics GmbH, Wedel, Germany) ensuring compliance with International Organization for Standardization (ISO) guidelines [20]. The IOLs were immersed in a balanced salt solution within a wet cell and aligned with a microscope objective and a charged-coupled device (CCD) camera. First, the effective focal length (EFL) was determined using the magnification method described in the ISO standard, utilizing monochromatic (546 nm) light at room temperature. The refractive power was calculated as the inverse of the EFL (*P* = 1/EFL). In the next step, image quality assessment was performed by means of the modulation transfer function (MTF) and the point spread function (PSF). To simulate clinical performance, measurements were performed using a model cornea inducing 0.13 μm of SA at a 5.15 mm diameter. The + 0.13 μm SA model cornea was selected as it represents a commonly used alternative to the ISO-standard + 0.28 μm configuration. From a clinical standpoint, published population data suggest considerable variability in corneal SA, with the SA range observed in the population from + 0.055 to + 0.544 μm [21]. Moreover, a recent population study highlights the age dependency of corneal SA, showing lower values in younger individuals [22]. The 0.13 μm model cornea therefore represents the lower end of the physiological range and is consistent with corneal SA values observed in younger or less aberrated eyes [23]. A spectral filter approximating the Commission Internationale de l’Éclairage (CIE) photopic luminosity function was applied. The optical quality was assessed at aperture sizes of 3.0 mm and 4.5 mm. Once the point of best focus was established via maximum MTF, through-focus curves were generated over a defocus range of + 1.0 D to -3.0 D at the IOL plane (equivalent to + 0.75 D to -2.25 D at the spectacle plane). At every focal step, the sagittal and tangential MTFs were averaged. Additionally, resolution was visualized using 1951 USAF test charts recorded at the 3.0 mm aperture. The area under the MTF (MTFa) was calculated to quantify overall optical quality, integrating spatial frequencies from 1 to 50 lp/mm following the ANSI Z80.35-2018 recommendations.

### Unwanted visual effects

To evaluate the potential for unwanted visual effects such as halos or glare, we analyzed the PSF using a 0.1-mm pinhole target at a 4.5-mm aperture. The choice of a 4.5 mm pupil for PSF evaluation was motivated by its clinical relevance for mesopic conditions, where unwanted photic phenomena (halos, glare) are most commonly perceived by patients. Additionally, the 4.5 mm aperture represents a condition where both the central Bessel-generating zone and the peripheral aspheric correction zone of the Extend IOL (which transitions at approximately 3.5 mm) are captured, providing the most comprehensive assessment of the overall light distribution. To visualize low-intensity light distribution (spurious images) beyond the PSF core, the dynamic range of the 8-bit camera was numerically extended by compositing images taken at varying shutter speeds [4, 24]. PSF images are presented at two saturation levels. The “optimally saturated” images display the central PSF core without pixel clipping, allowing comparison of the central peak intensity and immediate surround. The “maximum saturation” images deliberately overexpose the core to reveal the low-intensity peripheral light distribution (halos, scatter patterns) that would otherwise be invisible due to the limited dynamic range of the 8-bit camera. This technique of compositing multiple exposure levels to extend dynamic range is well-established in IOL PSF assessment [4].

To isolate the IOL design geometry from confounding aberration factors, this specific assessment was performed in monochromatic green light using model corneas matched to the specific spherical aberration correction of each lens (Extend: matched with 0.13 μm SA; ICB00: matched with 0.28 μm SA). The resulting light intensity profiles were plotted logarithmically against the visual angle.

### Data analysis

The analysis of optical-quality data and images was performed with custom-made software developed in MATLAB (MathWorks, USA).

## Results

### Power measurements

All the studied IOLs had their nominal powers labelled according to the ISO standard. Table 1 shows the nominal-power results presented as a mean ± SD (standard deviation).

**Table 1:** Measured nominal powers of the studied IOL models. SD = standard deviation

	Extend (Hanita)	ICB00 (J&J)
Mean [D]	20.32	19.76
SD [D]	0.06	0.06

### MTF metrics and USAF-chart images

**Fig. 2:**
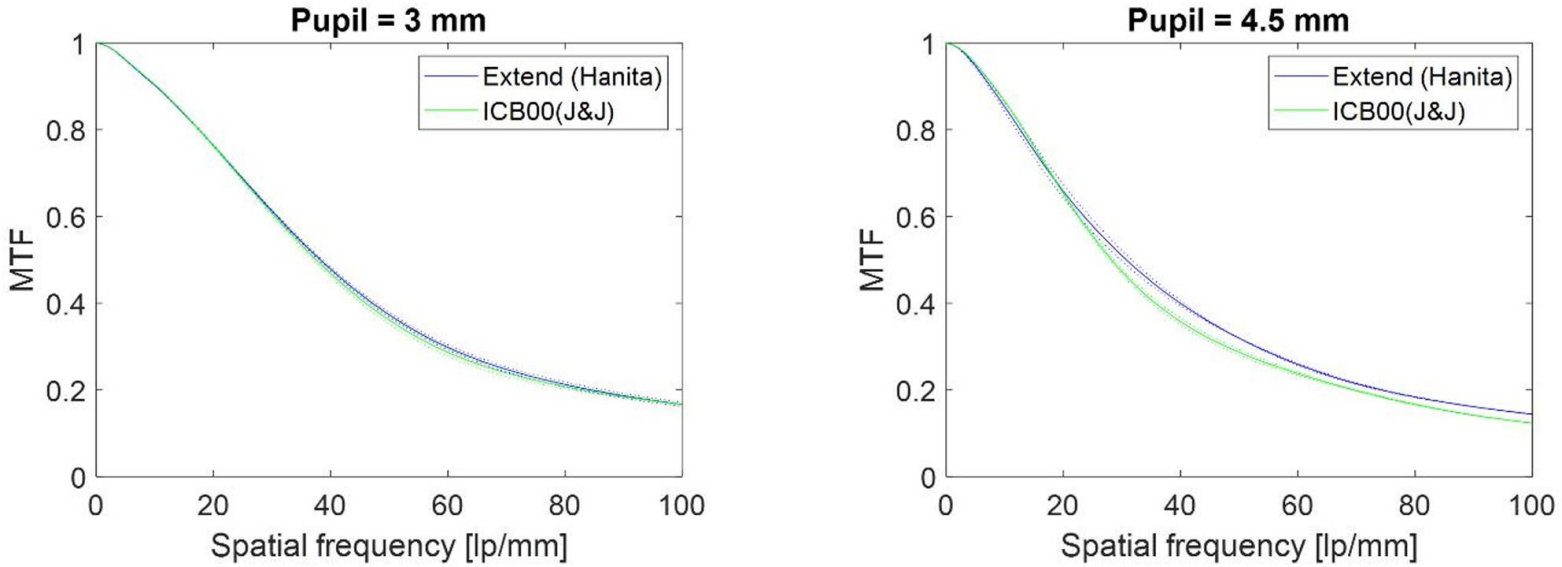
MTF levels of the studied IOLs at the best focus for 3- and 4.5-mm apertures. The dotted lines show the values of each lens separately; the solid lines refer to the average of two IOLs

Figure 2 presents the MTF curves of all IOL samples measured at the best focus through the 3- and 4.5-mm apertures.

At 3 mm, both the Extend and the ICB00 demonstrated comparable optical performance. The Extend IOLs produced an average MTF @ 50 lp/mm of 0.38 ± 0.01, while for the ICB00, it was 0.36 ± 0.01.

At 4.5 mm, the Extend’s MTF was 0.32 ± 0.00, which was minimally higher than that of the ICB00 (0.29 ± 0.00) at 50 lp/mm. However, for lower spatial frequencies, the two models were comparable.

**Fig. 3:**
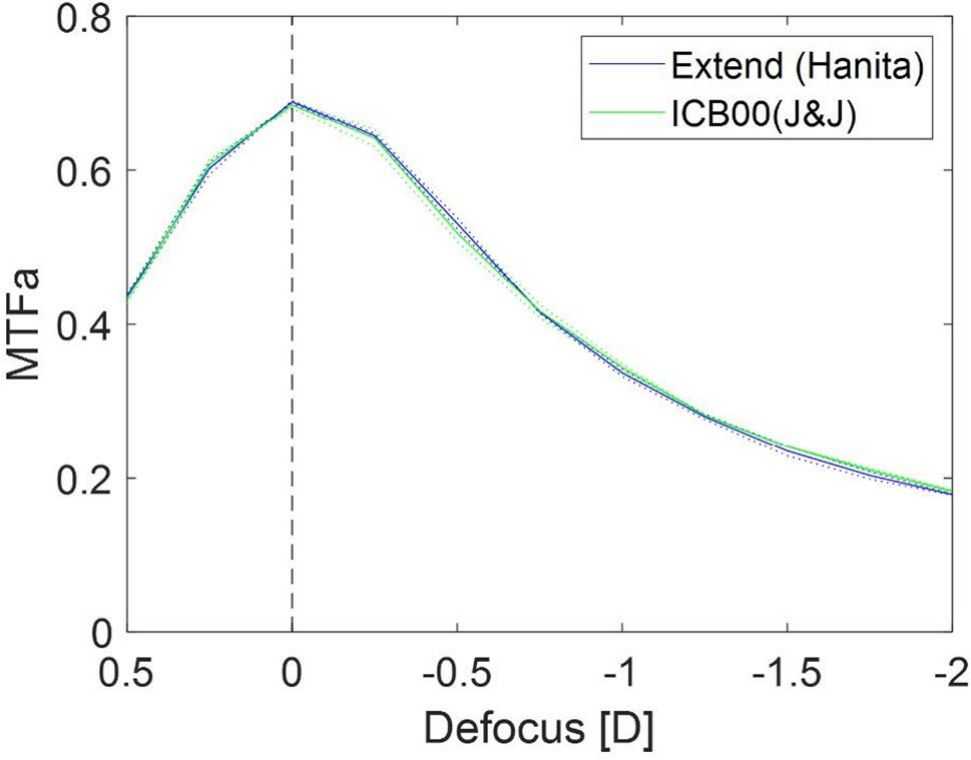
The MTFa curve of the two studied IOLs measured at the defocus range from + 0.5 D to -2 D at the spectacle plane

Figure 3 reports the MTFa change for the enhanced-monofocal IOLs. The MTFa of the Extend and the ICB00 were virtually the same for studied defocus, showing one peak extended towards negative (intermediate-range) values.

**Fig. 4:**
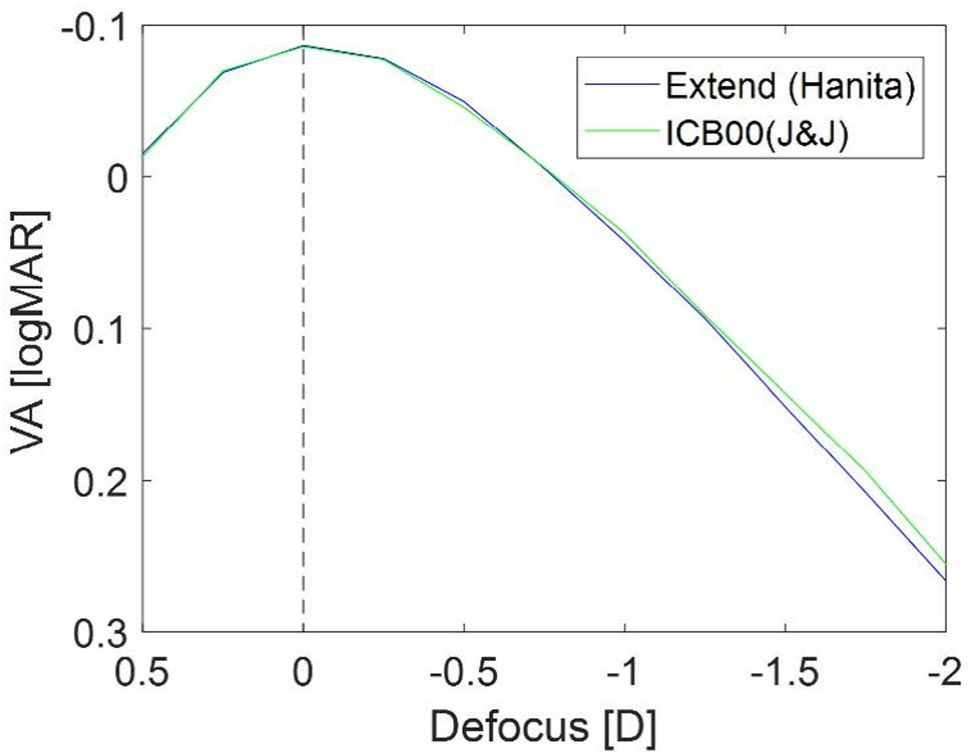
The logMAR VA of three studied IOLs measured at the defocus range from + 0.5 D to -2 D (spectacle plane). The dotted lines show each lens’ values separately; the solid lines refer to the average of two samples

LogMAR VA (Fig. 4) was derived from the MTFa according to the empirical model as described in the Materials and Methods section. The Extend and the ICB00 yielded identical VA at far focus (-0.09 logMAR). Although for lower defocus. i.e., less than − 1.25 D, the two curves show a nearly complete overlap; above this level, the ICB00 appears to produce minimally better VA. However, the observed difference accounts for < 0.02 logMAR, which is below the accuracy level of standard VA testing.

**Fig. 5:**
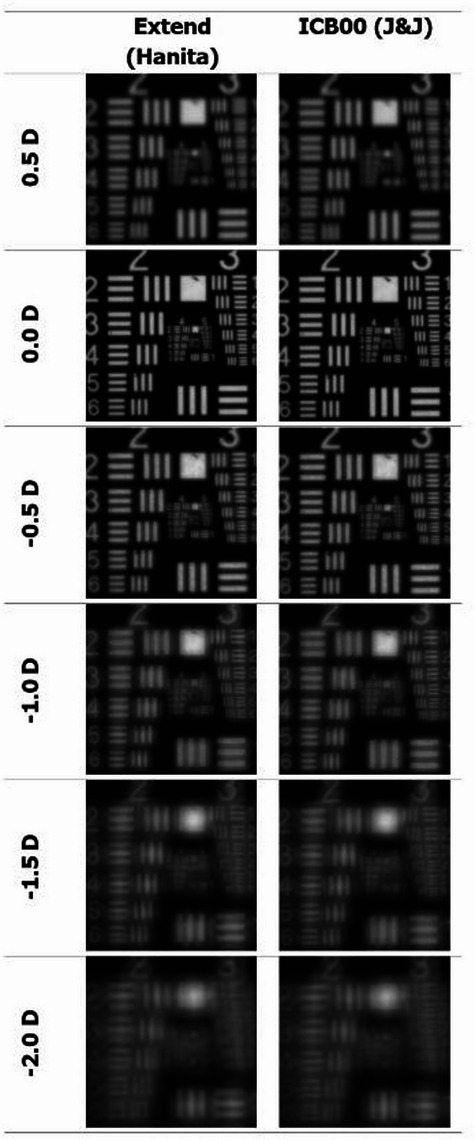
USAF-resolution targets recorded at a defocus range of + 0.5D to -2.0D (spectacle plane) and the 3-mm aperture

The resolution-test images presented in Fig. 5 confirm the comparable MTFa results of the two models.

### PSF assessment

Table 2 presents the PSF images captured at two different exposure levels. These images present the visualization of dysphotopsia. At optimal saturation, the central PSF core was well-resolved for both IOL models, allowing direct comparison of peak intensity distribution. At maximum saturation, the deliberately overexposed core revealed low-intensity peripheral light patterns that would otherwise fall below the camera’s dynamic range. The Extend IOL displayed a minimally broader halo pattern compared to the ICB00.

**Table 2:**
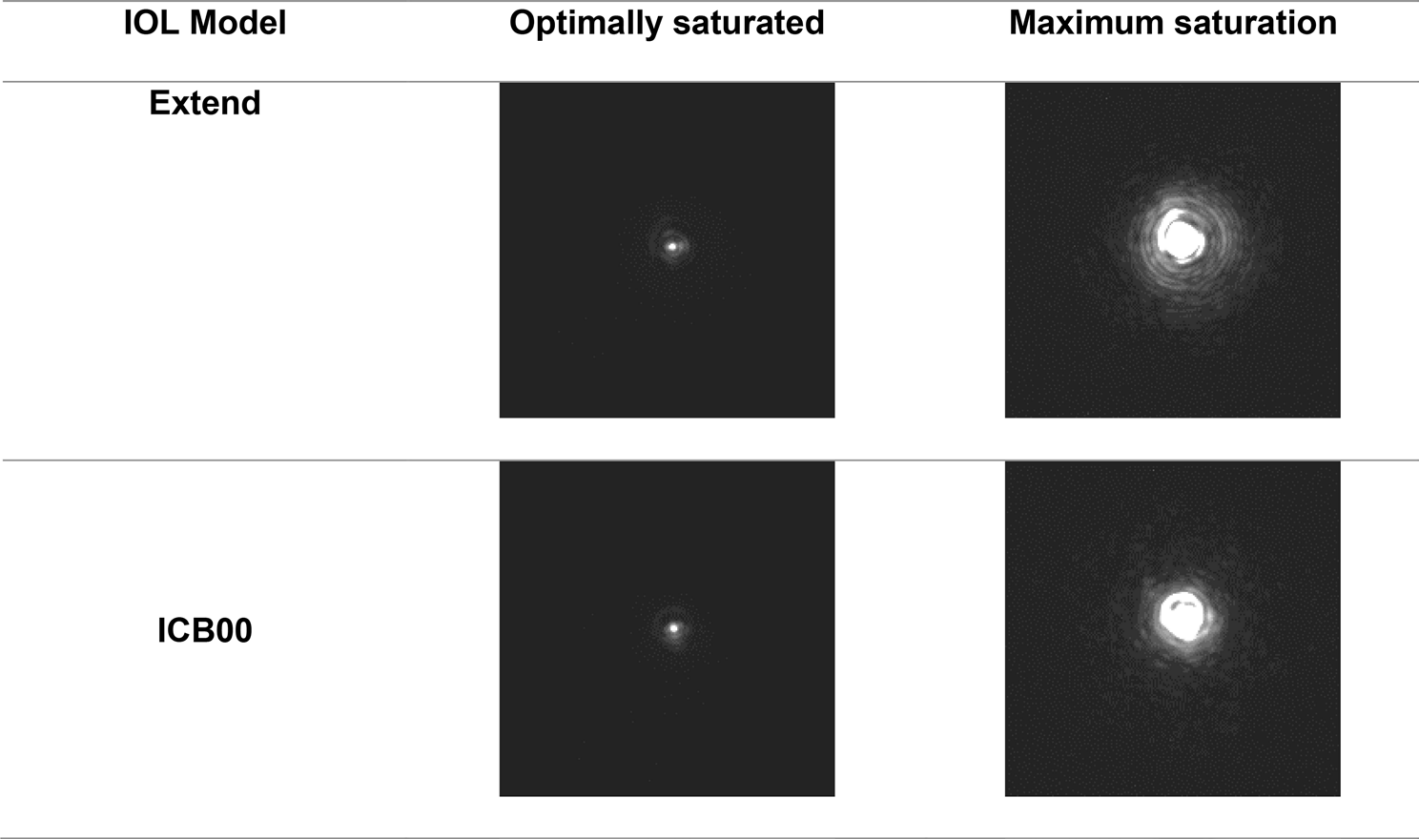
Point spread function images of the two studied IOL models at a 4.5 mm pupil diameter. The left column shows optimally saturated images resolving the central PSF core. The right column shows maximally saturated (overexposed) images, revealing low-intensity peripheral light distribution beyond the core

**Fig. 6:**
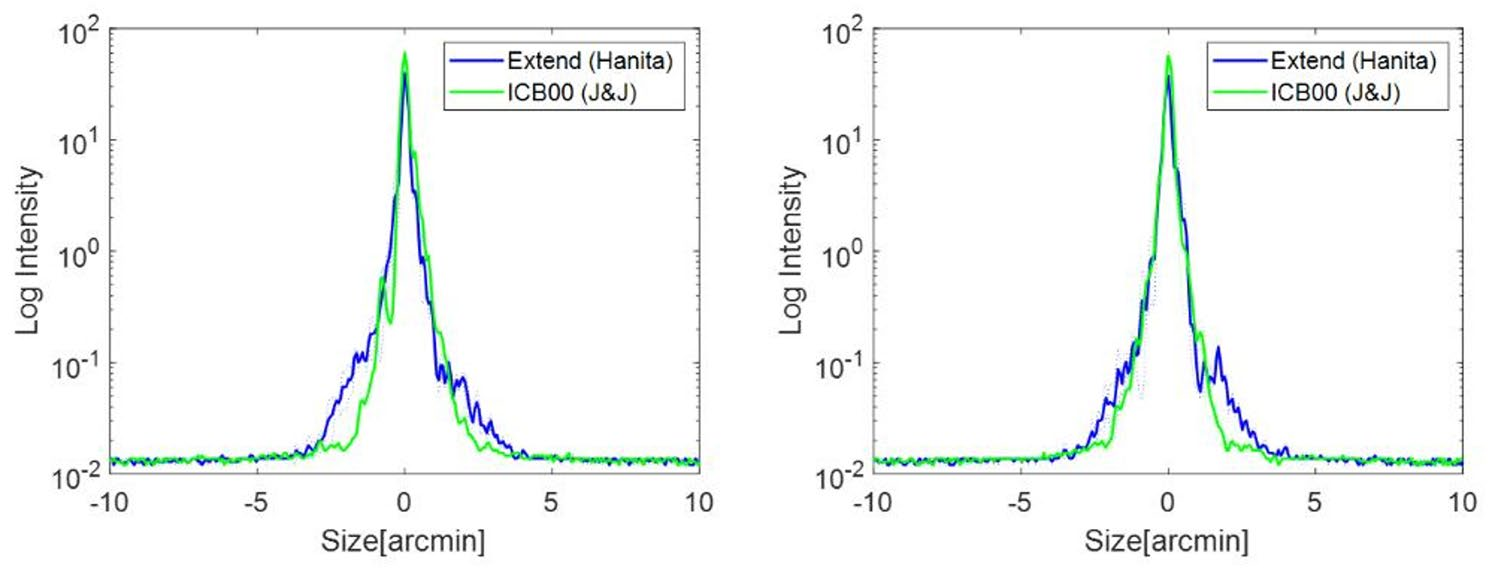
The horizontal (left panel) and vertical (right panel) cross-section of the PSF’s intensity profile of the two IOL models. The dotted lines show each lens’ values separately; the solid lines refer to the average of two samples

Figure 6 demonstrates the PSF’s horizontal and vertical cross-sections of the two models. This figure shows the accurate and numerical results corresponding to the visualization of Table 2. The estimated extent of light spread, as shown in Fig. 6, was 5.6 ± 0,14 arcmin and 3.9 ± 0,14 arcmin for the Extend and the ICB00. Overall, the Extend and the ICB00 presented a similar light-intensity decline with a slight and localized increase at about 2 arcmins seen with the Extend IOLs.

## Discussion

This study demonstrates that the Extend IOL provides a good optical performance comparable to the established ICB00 IOL. The two models showed nearly equivalent MTF levels at the 3-mm pupil as well as lower defocus values. Although minimal differences in the MTFa and predicted VA were observed at higher defocus, it was estimated to be < 0.02 logMAR. One line on the ETDRS chart corresponds to 0.1 logMAR and the repeatability of clinical VA measurements typically exceeds the 0.02 logMAR level. Therefore, a difference of less than 0.02 logMAR is small and considered to be below the resolution threshold of standard clinical VA testing.

The large amount of data available for the ICB00 shows a good agreement with our laboratory prediction. Studies show a monocular depth of focus of 1.23 D for ICB00 compared to around 0.9 D for standard monofocal lenses, with an increase of 2–5 ETDRS letters at levels of -1.25 and − 1.5 D. The defocus curve usually shows a VA of 0.2 logMAR or better from 0 D to approximately − 1.5 D both in monocular and binocular conditions [8, 15, 17]. The clinical data for the ICB00 also extends towards populations of eyes with comorbidities, which show that the IOL is generally suitable for these cases [12, 13]. As mentioned, there is no clinical data available for the Hanita Extend as of today. Given the optical similarities shown in our laboratory analysis, we can hypothesize that both lenses should also have similar optical qualities in a clinical/real-world setting.

Clinical data on the ICB00 continuously shows a level of dysphotopsias that is comparable to standard monofocal IOLs. Halo and Glare are very rarely reported [16, 17]. Looking at the PSF-Analysis one notices that depending on the cross-section, the PSF of the Extend does appear to be slightly elevated compared to the Eyhance. These findings still greatly differ from PSF-analysis found in EDoF or trifocal IOLs [25, 26], so one may question whether these minute differences will translate into a tangible clinical difference between the two monofocal+ IOLs [9]. We suspect the Extend IOL to have a similar dysphotophsia profile compared to the ICB00. This of course necessitates clinical validation of our laboratory findings.

An interesting point to discuss is why the Eyhance ICB00 is not labelled as an EDoF-IOL. IOL Classification has been an important topic of discussion ever since models with an increased focus range were first introduced [27]. This was further expanded with the addition of monofocal+ IOLs, which are usually regarded as a separate category in between standard monofocal and EDoF IOLs. The ISO 11979-7, 2024 categorizes IOLs into 4 groups: monofocal, toric, simultaneous vision lenses (SVLs) and accommodating lenses. These SVLs are further classified into multifocal IOLs (mIOL), extended depth of field (EDF) and full visual range (FVR) which interestingly shows that monofocal+ lenses are not individually represented by ISO, but instead would (probably) be counted as monofocal IOLs [20].

In August 2024 the ESCRS (European Society of Cataract and Refractive Surgeons) Functional vision working group published an evidence-based nomenclature, that tried to improve distinction between EDoF and monofocal-plus IOLs based on clinical evidence for these IOLs. Monofocal+ would then be considered a partial-RoF IOL with enhanced RoF, defined as a RoF in 0.2 logMAR between 1.2 and 1.58 D, while an EDoF-IOL would have to prove a VA of 0.2 LogMAR from 1.58 to 2.3 D at most. Both models would show no VA increase from Intermediate to near [27].

Although in some patients, the ICB00 may reach a RoF that goes beyond 1.58 D and therefore be categorized into “extended” category [27], only the consistency of this effect, confirmed through randomized clinical trials, can determine its accurate classification. It is of great importance for all further classifications and improvements that the intrasubject variability due to aberrations and pupil size variation is kept in mind, when defining strict numerical cutoffs [28–31].

Apart from their comparable optical and thus expected visual function, these two lenses share other similarities: both IOLs are C-Loop models that have an optical and overall diameter of 6 and 13 mm, respectively. Furthermore, both lenses are made out of a hydrophobic acrylic material that block violet and ultraviolet rays of light. Another feature both IOLs share are the squared 360° edges. Both IOLs are offered from + 5 to + 34 D, though the Hanita Extend currently offers only 1 D increments for IOL powers of + 30 D and above. It may also be noted that the Hanita Extend has a different A-constant for optical biometry or ultrasound for preoperative IOL calculation.

Regarding the limitations of this study, it is important to acknowledge that the in vitro conditions (BSS immersion, room temperature, ISO-model cornea) represent a controlled simplification that enables standardized, reproducible comparison between IOL designs, while real-world optical performance is modulated by individual ocular biometry, physiological temperature, pupil dynamics, and neuroadaptation. We note that our laboratory has previously shown good agreement between bench-derived performance predictions and clinical outcomes for multiple IOL designs, lending confidence to the translational value of these measurements, while acknowledging that clinical validation remains essential.

One of the more specific study limitations is that the corneal model selected favors the Extend IOL, as it closely aligns with its SA correction and thus improves its performance at 4.5 mm compared with the ICB00. However, it is important to note that ISO 11979-2 allows such a corneal model to be used in IOL testing, and this type of model cornea is a commonly used configuration in optical bench studies and has been employed in prior peer-reviewed publications from other centers [32]. Moreover, + 0.13 μm SA lies well within the broad range of SA values reported clinically [21, 23, 33]. In addition, a recent large-population study indicated that SA is an age-dependent variable, with lower values in younger healthy eyes and higher values in older eyes [22], in which an average of approximately + 0.28 μm is often reported in cataract-age populations [34]. The MTF and predicted VA results were obtained under aforementioned SA condition and may not fully generalize to all corneal aberration profiles encountered clinically.

It is important to note that another limitation is that PSF testing was only conducted at a pupil size of 4.5 mm. Though we provided reasoning for this decision, additional pupil sizes would further characterize the pupil dependent behaviour of the Bessel-like optic and should be investigated in future studies. Both IOLs differ in Abbe number, which means that the Hanita Extend has marginally higher material dispersion and thus potentially more longitudinal chromatic aberration. While MTF/MTFa measurements were conducted under polychromatic light, PSF testing was conducted under monochromatic light conditions. This was done deliberately to isolate the geometric design features and eliminate chromatic confounders. This results in a limitation regarding the translation of our PSF results to the polychromatic clinical/real-world environment.

This study did not evaluate the effect of clinically relevant tilt and decentration on the optical performance of these IOLs, which is a clear limitation. These effects should be evaluated in future studies, using already established methodology [35].

## Conclusion

The Hanita Extend IOL shows comparable results to the Tecnis Eyhance ICB00 in a a laboratory setting regarding MTF function, USAF-Chart images and the PSF assessment. Using these metrics, both IOLs showed comparable optical performance, as well as laboratory-derived VA, visual range and amount of dysphotopsia. These findings suggest that the Hanita Extend IOL has the potential to provide similar visual outcomes – a hypothesis that still requires validation through prospective clinical studies.

## Data Availability

The datasets used and/or analysed during the current study are available from the corresponding author on reasonable request.

## Abbreviations

ANSI: American National Standards Institute
CCD: Charge-coupled device
CIE: Commission Internationale de l’Éclairage (International Commission on Illumination)
D: Dioptre(s)
EDF / EDoF: Extended Depth of Field / Focus
EFL: Effective Focal Length
ESCRS: European Society of Cataract and Refractive Surgeons
ETDRS: Early Treatment Diabetic Retinopathy Study (standardized eye charts)
FVR: Full Visual Range
IOL: Intraocular Lens
ISO: International Organization for Standardization
logMAR: Logarithm of the Minimum Angle of Resolution
lp/mm: Line pairs per millimeter
mIOL: Multifocal Intraocular Lens
MTF: Modulation Transfer Function
MTFa: Area under the MTF (Modulation Transfer Function area)
PSF: Point Spread Function
RoF: Range of Focus
SA: Spherical Aberration
SD: Standard Deviation
SVL: Simultaneous Vision Lenses
USAF: United States Air Force (referring to the 1951 USAF test chart)
VA: Visual Acuity
